# Spatial, geographic, and demographic factors associated with adolescent and youth suicide: a systematic review study

**DOI:** 10.3389/fpsyt.2024.1261621

**Published:** 2024-02-09

**Authors:** Masoud Ghadipasha, Ramin Talaie, Zohreh Mahmoodi, Salah Eddin Karimi, Mehdi Forouzesh, Masoud Morsalpour, Seyed Amirhosein Mahdavi, Seyed Shahram Mousavi, Shayesteh Ashrafiesfahani, Roya Kordrostami, Nahid Dadashzadehasl

**Affiliations:** ^1^ Legal Medicine Research Center, Legal Medicine Organization, Tehran, Iran; ^2^ Department of Gastroenterology and Hepatology, School of Medicine, Shahid Modarres Hospital, Shahid Beheshti University of Medical Sciences, Tehran, Iran; ^3^ Social Determinants of Health Research Center, Alborz University of Medical Sciences, Karaj, Iran; ^4^ Social Determinants of Health Research Center, Health Management and Safety Promotion Research Institute, Tabriz University of Medical Sciences, Tabriz, Iran; ^5^ Department of Criminal Law and Criminology, Islamic Azad University, Tehran, Iran

**Keywords:** spatial analysis, geography, suicide, adolescents, youth, systematic review

## Abstract

**Background:**

Suicide is a public health issue and a main cause of mortality among adolescents and the youth worldwide, particularly in developing countries.

**Objectives:**

The present research is a systematic review aiming to investigate the spatial, geographical, and demographic factors related to suicide among adolescents and the youth.

**Methods:**

In this systematic review, two researchers examined PsycINFO, Web of Science, Scopus, and PubMed databases on December 7^th^, 2022 with no time limits from the beginning of publication until 2022 to identify the primary studies on spatial and geographic analysis on adolescent and youth suicides. Once duplicate studies were identified and removed, the titles and abstracts of studies were examined and irrelevant studies were also removed. Finally, 22 studies were reviewed based on the inclusion criteria.

**Results:**

Our findings show that suicide rates are generally higher among men, residents of rural and less densely populated regions, coastal and mountainous regions, natives, 15-29 age group, less privileged populations with social fragmentation, unemployed, divorced or lonely people, those who live in single parent families, people with mental health issues, and those with low levels of education.

**Conclusions:**

Stronger evidence supports the effects of geographic and demographic variables on youth and adolescent suicide rates as compared with spatial variables. These findings suggest that policy makers take spatial and demographic factors into consideration when health systems allocate resources for suicide prevention, and that national policymakers integrate demographic and geographic variables into health service programs.

**Systematic Review Registration:**

https://www.crd.york.ac.uk/prospero/, identifier CRD42023430994.

## Backgrounds

Suicide is defined as a death directly or indirectly caused by intentionally poisoning or injuring oneself (1). As a serious public health issue, suicide constitutes the cause of death of about 800,000 (1.4%) individuals per year in the world (2). Approximately 78% of suicide cases have been reported in low-income countries (3). The annual frequency of suicide in different countries ranges from below 1 per 100,000 deaths in Saudi Arabia and Belize to over 40 per 100,000 deaths in Lithuania and Guyana (4, 5). In 2016, the WHO estimated the annual mortality from suicide at 10.7 per 100,000 (6).

Suicide is a major cause of mortality among the youth and adolescents, especially in developing countries (7). In 2015, suicide was reported as the cause of death among 6% of adolescents (8, 9). After road accidents, suicide constitutes the second leading cause of death among individual aged 10-24 years (10, 11). Research suggests 1-10% of adolescents commit suicide at least once in their life given the social stigma of suicide and its misclassification (12–14), the suicide frequency is underestimated at 164,000 in individuals aged below 25 years (15, 16).

Pesticide poisoning, hanging and use of firearms globally constitute the cause of 30% of suicides. The means of suicide used by the victims largely depends on their accessibility to lethal objects (17, 18). Suicide exerts severe and long-lasting effects on the family and friends as suicide survivors. Research suggests positive relationships between degree of depression in the bereaved and their closeness with those committing suicide (19, 20). “Suicide and self-inflicted injuries” was the 14th and 18th (21) leading cause of disability-adjusted life years in 2013 and 2016, respectively (22).

Given the significant social and individual effects of suicide, acquiring awareness of its temporal and spatial patterns in different demographic groups by age, gender and ethnicity and identifying the causes of changes in these patterns are essential for designing effective suicide control and prevention plans. Identifying both risk factors and socio-geographical background is also integral to an effective suicide prevention strategy (23, 24).

Spatial analysis can help investigate the geographic pattern of suicide (25), identify areas with greater risk of suicide, explore the potential relationship between local factors and suicide risk (26), and assess the rates across geographic units (27).

Multiple factors are associated with suicide as the outcome of complex interactions of individuals with family members and their community (28). Research suggests suicide relates to genetic, social and family factors (29) and psychological factors such as depression and anxiety (29–31) as well as adverse childhood experiences, neglect by parents (32), age, gender, sexual orientation, socioeconomic status (33, 34), academic achievement and absenteeism (35, 36), and substance abuse (2). Suicide protective factors also include having a large number of children, family support, coping skills and religiousness (37–39).

Despite the importance of these studies, their limitations include failure to explain suicide and its distribution in different locations. National and global initiatives based on early risk detection and management play a key role in saving lives and suicide prevention as a public health priority. As a suicide monitoring method, spatial and geographic analyses have been conducted to identify high suicide-risk areas (40). These analyses can assist policymakers in determining the causes of suicide, predicating local suicide patterns based on suicide-related data and developing suicide prevention strategies and appropriate interventions in high-risk regions. The present research was therefore conducted to systematically review the spatial and geographic analysis of suicide and its demographic factors in adolescents and the youth.

## Methods

### Study design

This systematic review was performed to investigate the spatial, geographic and demographic factors of suicide in adolescents and the youth based on the Preferred Reporting Items for Systematic Reviews and Meta-Analyses (PRISMA) (41), as a guideline for appropriate and accurate information sources. After formulating the research question, the search strategy was designed and the systematic review was conducted by screening for eligible articles. Afterwards, two researchers independently employed the Strengthening the Reporting of Observational studies in Epidemiology (STROBE) (42) to qualitatively evaluate the articles and extract data. A third person resolved potential conflicts in the interpretation of data. This systematic review has been registered on the International Prospective Register of Systematic Reviews (PROSPERO, Registration number: CRD42023430994).

### Research question

The research question was formulated based on the population (P), exposure (E), comparator (C), and outcome of interest (O) in the review (PECO) for spatial and geographic analysis of completed suicide in teenagers and young adults (Studies and findings related to suicidal idea and suicidal thoughts, suicidal intention, unsuccessful attempts to commit suicide were excluded). PECO helps researchers create research questions (43). Three main dimensions of spatial, geographic, and demographic factors (E, exposure, interest), suicide (O, outcome based on the study interest) of adolescent and young boys and girls, and (P, population) were investigated by the researchers. Accordingly, what is the research question, and spatial, geographic, and demographic factors related to suicide in adolescents and the youth? It must be noted that the study was not context-specific (C).

### Systematic search

**Table 1** presents the strategy of systematic search before identification and screening. The keyword search was enriched in the identification stage using synonyms and based on MeSH in PubMed, and was modified for other databases. Boolean operators were also used along with keywords. We retrieved 3001 articles in the systematic literature search conducted in PubMed, Scopus, Web of Science and PsycINFO on 7 December 2022. These four databases were selected due to their academic nature and accessibility in Iran. The retrieved data were entered into EndNote and 1040 duplicate articles were identified and eliminated.

**Table 1:** Keyword search used in the identification process.

**PubMed**	(Suicide*[tiab] OR “Attempted Suicide*”[tiab] OR “Suicide Attempt”[tiab] OR Parasuicide*[tiab] OR “Completed Suicide*”[tiab] OR “Fatal Attempt*”[tiab] OR “Fatal Suicide*”[tiab]) AND (spatial[tiab] OR “Spatial Analyses”[tiab] OR “Spacial Analysis”[tiab] OR “Spacial Analyses”[tiab] OR Kriging*[tiab] OR “Spatial Interpolation*”[tiab] OR “Spatial Autocorrelation*”[tiab] OR “Spatial Dependenc*”[tiab] OR “Kernel Density Estimation*”[tiab] OR “spatial regression”[tiab] OR “Geographically Weighted Regression*”[tiab] OR “Geographic Mapping*”[tiab] OR “Geographic Cartography”[tiab] OR “Dasymetric Mapping*”[tiab] OR Geocoding[tiab] OR “Choropleth Mapping*”[tiab] OR Georeferencing[tiab] OR spacial[tiab] OR geographic*[tiab] OR cluster*[tiab]) AND (child*[tiab] OR adolescence[tiab] OR adolescent*[tiab] OR youth*[tiab] OR teen*[tiab] OR teenager*[tiab])
**Scopus**	TITLE-ABS-KEY(suicide* OR “Attempted Suicide*” OR “Suicide Attempt” OR Parasuicide* OR “Completed Suicide*” OR “Fatal Attempt*” OR “Fatal Suicide*”) AND TITLE-ABS-KEY(spatial OR “Spatial Analyses” OR “Spacial Analysis” OR “Spacial Analyses” OR Kriging* OR “Spatial Interpolation*” OR “Spatial Autocorrelation*” OR “Spatial Dependenc*” OR “Kernel Density Estimation*” OR “spatial regression” OR “Geographically Weighted Regression*” OR “geographic mapping*” OR “Geographic Cartography” OR “Dasymetric Mapping*” OR Geocoding OR “Choropleth Mapping*” OR Georeferencing OR spacial OR geographic* OR cluster*) AND TITLE-ABS-KEY(child* OR adolescence OR adolescent* OR youth* OR teen* OR teenager*)
**Web Of Science**	TS=(suicide* OR “Attempted Suicide*” OR “Suicide Attempt” OR Parasuicide* OR “Completed Suicide*” OR “Fatal Attempt*” OR “Fatal Suicide*”) AND TS=(spatial OR “Spatial Analyses” OR “Spacial Analysis” OR “Spacial Analyses” OR Kriging* OR “Spatial Interpolation*” OR “Spatial Autocorrelation*” OR “Spatial Dependenc*” OR “Kernel Density Estimation*” OR “spatial regression” OR “Geographically Weighted Regression*” OR “geographic mapping*” OR “Geographic Cartography” OR “Dasymetric Mapping*” OR Geocoding OR “Choropleth Mapping*” OR Georeferencing OR spacial OR geographic* OR cluster*) AND TS=(child* OR adolescence OR adolescent* OR youth* OR teen* OR teenager*)
**PhsycINFO**	(suicide* OR “Attempted Suicide*” OR “Suicide Attempt” OR Parasuicide* OR “Completed Suicide*” OR “Fatal Attempt*” OR “Fatal Suicide*”) AND (spatial OR “Spatial Analyses” OR “Spacial Analysis” OR “Spacial Analyses” OR Kriging* OR “Spatial Interpolation*” OR “Spatial Autocorrelation*” OR “Spatial Dependenc*” OR “Kernel Density Estimation*” OR “spatial regression” OR “Geographically Weighted Regression*” OR “geographic mapping*” OR “Geographic Cartography” OR “Dasymetric Mapping*” OR Geocoding OR “Choropleth Mapping*” OR Georeferencing OR spacial OR geographic* OR cluster*) AND (child* OR adolescence OR adolescent* OR youth* OR teen* OR teenager*)

#### Screening and inclusion and exclusion criteria

Two researchers separately assessed the titles and abstracts of 1961 studies for relevance. The search was run from database inception till December 7^th^, 2022 with no time limits with the inclusion criteria of being an original study in the English language, and focused on the spatial and geographic analysis of suicide in adolescents and the youth. Only studies on completed suicides were included. Studies and findings for suicidal ideation, suicide attempts, and failed suicides were excluded. Thus, qualitative studies, case reports, systematic reviews, meta-analyses, review studies, conference papers, book chapters, letters to editors, and intervention studies were excluded. Finally, 82 studies remained for full text evaluation.

### Eligibility

**Figure 1:**
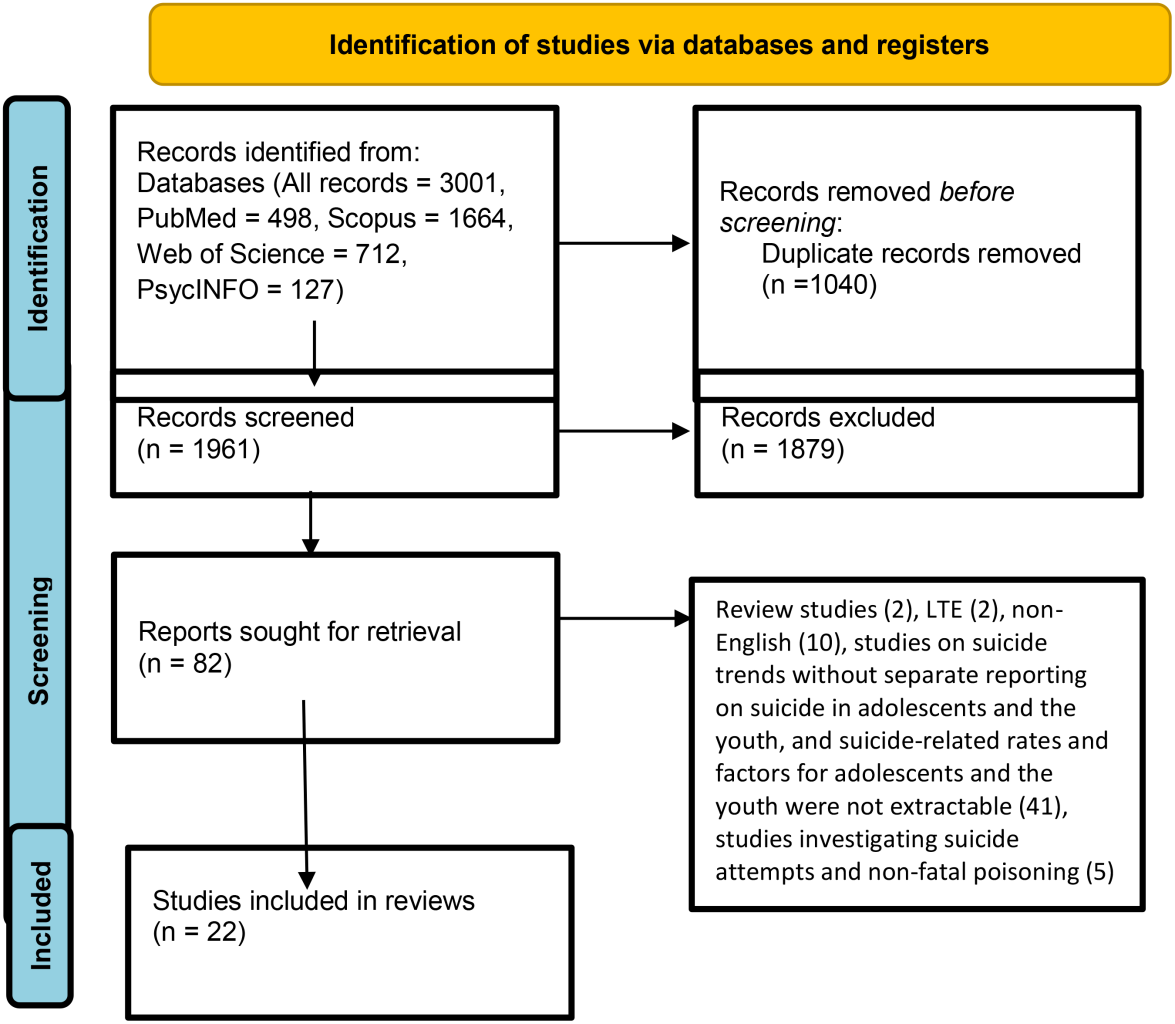
PRISMA flow diagram.

Two of the authors independently evaluated the full texts of 82 studies, which resulted in the exclusion of 60 studies for reasons stated in **Figure 1**. The remaining studies were entered into the quality evaluation and data extraction process.

### Quality assessment

The remaining articles were examined after the full-text reading stage and determining the eligibility in terms of the risk of bias assessment to ensure the quality of the studies (44). The quality of the studies was evaluated based on the STROBE checklist, and finally 22 articles were included for review in the present study. Quality assessment was performed by two people independently. The articles that scored between 11-16 were evaluated as appropriate and the articles that scored more than 16 were evaluated as good (45, 46). Each study was evaluated to check the potential risk of biases through the key areas of study design, sample size justification, target population, sampling strategy, sample selection, validity and reliability of measurement, methodological limitations, and discussion. Any differences between the two authors were resolved through discussion until an agreement was reached.

The two researchers independently extracted the following data from the studies: author’s name, publication date, country, study objective, study design, sample volume, statistical test, and findings. Disputes were resolved by talking and help from a third person. In the next stage, the researchers systematically merged the findings based on the use of words, text, and study findings to explain relationships between the extracted data (47). Discussions between the researchers about the relationship of similar data led to classification of the data into different categories. This technique was repeated until logical findings were formed for interpretation. **Table 2** presents the results of the study review.

**Table 2:** Data extraction and evaluation of study quality.

No	Author (Year), Country	Objective	Study Design	Data source	Sample size	Suicide rate	Statistical test	Findings	Quality assessment score
**1**	Hajebi, et al, 2016, Iran, data from 2009-2012 (48)	To study the trend, correlations and discrepancy of registered suicide incidents in Iran	Descriptive	Universities of medical sciences and health services (UMSs) via their health network centers in each district, city or town	4879	5-14 = 2.7, 15-25 = 2, 25-34 = 2.7	Logistic regression analysis	Attempted suicides showed more fatality in males, older adults, widows/widowers, divorced and unemployed subjects as well as in residents of rural areas.	16
**2**	Ivey-Stephenson, et al, 2017, USA, data from 2001-2015 (49)	To examine annual county level trends in suicide rates during 2001–2015 among and within urbanization levels by select demographics and mechanisms of death	Retrospective	National Vital Statistics System	71306	10-14 = 1.36, 15-24 = 10.42	Joinpoint regression analyses	Suicide rates increased across the three urbanization levels, with higher rates in nonmetropolitan/rural counties than in medium/small or large metropolitan counties. Across urbanization levels, suicide rates were consistently highest for men and non-Hispanic American Indian/Alaskan natives compared with rates for women and other racial/ethnic groups; however, rates were highest for non-Hispanic whites’ person, in more metropolitan counties. Trends indicate that suicide rates for non-Hispanic black person were lowest in nonmetropolitan/rural counties and highest in more urban counties.	17
**3**	Diego Salmero´ n, et al, 2013, Spain, data from, 1991-2008 (50)	To analyze the trends, geographical variations, seasonal patterns and methods of mortality due to the combination of suicide and causes of undetermined intent in Spain	Retrospective	The National Statistics Institute	13985	0-14=.18, 15-24 = 4.51, 25-34 = 7.21	Poisson models	Spring and summer were the seasons with the highest suicide rates.	17
**4**	IB O’Farrell, et al, 2016, Ireland, data from 2009-2011 (23)	To examine the small area level association between suicide and the following three area level factors, deprivation, social fragmentation and population density	Retrospective	The Irish Central Statistics Office	781	All=15, males=25, females= 5	Negative binomial regression	The most deprived areas showing the greatest risk of suicide. Low population density (rurality) was associated with an increased risk suicide in males. A weak association between high population density (urbanicity) and increased suicide risk was found among females in the 15–39-year age group.	16
**5**	Xin Qi, et al, Australia, 2014, data from, 1986-2005 (51)	This study explores the spatiotemporal variations of suicide across Australia	Retrospective	Australian Bureau of Statistics	18301	15-34 male=28.29, female=6.12	Descriptive and mapping approaches	Differences in suicide rates across genders were found across geographical areas.	17
**6**	Pompili, et al, 2008, Italy, data from 19970-2002 (52)	To analyze gender and regional differences in the suicide rate of adolescents	Retrospective	Italian Mortality Database, which is collected by the Italian National Census Bureau	3069	2.35	Poisson regression	Analyses of these suicides identified significant differences by region of residence and gender.	16
**7**	Song, et al, 2019, South Korea, data from2005-2015 (53)	To investigate the influence of area-level factors on adolescent suicide and to determine which variables differ according to age and gender	Retrospective	Korean Statistical Information Service and the Korea Labor Institute		10-14 = 1.41, 15-19 = 8.19	A panel data model using Generalized Least Squares	Economic problems were shown to be more associated with suicide in male adolescents than in female adolescents. On the other hand, social fragmentation and health services were shown to be more associated with suicides in females	17
**8**	Middleton, et al, 2006, England and Wales, data from 1988-1994 (54)	To investigate the spatial patterning and possible contributors to the geographical distribution of suicide among 15–44- year-old men	Small-area analysis and mapping of geo-coded	Suicide and undetermined deaths (International Classification of Diseases	15821		Random-effects Poisson regression models	Suicide rates were highest in the inner-city areas, coastal areas, particularly those in more remote regions. Social fragmentation, such as the proportion of single-person households, is associated with rates of suicide in both urban and rural areas. Levels of unemployment and long-term illness accounted for some of the coastal patterning.	17
**9**	Cynthia A. Fontanella, et al, 2015, USA, data from 1996-2010 (55)	To examine trends in the US suicide mortality for adolescents and young adults across the rural-urban continuum	Retrospective	The National Center for Health Statistics National Vital Statistics System	66595	Urban=10.31, rural= 19.93	Negative binomial regression models	Youths died by suicide and rural suicide rates were nearly double those of urban areas for both males and females, rural place of residence.	17
**10**	Fong Ans yip, 2003, Hong Kong, data from, 1991-1996 (56)	To study the geographical distribution of suicides in Hong Kong and examine the influence of socioeconomic variables on suicides and delineate the etiological factors	Retrospective	All deaths in Hong Kong where the underlying cause was determined as being suicidal or self-inflicted injury (E950-E959)		.97	Pearson’s correlation coefficient	High population density and proportion of Cantonese residents	15
**11**	Gyung-Mee Kim, et al, south Korea, 2019, data from, 2001-2010 (57)	To determine the trends and the regional risk factors of youth suicide in South Korea from 2001 to 2010	Retrospective	National Statistical Office of South Korea (NSO)	2167	2001 = 18, 2010 = 47.04	T -tests, Chi squared test	No significant gender difference in suicide rates; there was a significantly higher ratio of adolescents aged 15-18 versus adolescents aged 12-14 and higher number of single-parent households than those in the low SMR regions, higher number of adolescents who were treated with depression were related to elevated suicide rates of adolescents. Total sleep time of adolescents and regional unemployment rates were negatively associated with the suicide risk of respective regions.	17
**12**	Núñez-González, et al, 2018, Ecuador data from 1997-2016 (8)	To describe the temporal trend of suicide in adolescents between 10-19 years old	Ecological study	National Institute of Statistics and Census (INEC) database	3824	1997 = 12.7, 2016 = 23.3	Pearson’s Chi squared test, the Fisher Exact test	No significant differences between the monthly distribution of suicides and gender, indigenous people in the Amazon region and the Southern Highlands.	17
**13**	BERATIS, 1991, Greece, data from 1980-1987 (58)	To examine epidemiological characteristics among these youngsters, and identify subgroups which appear to be at higher risk for suicide	Retrospective	The data were collected directly from the records of the police headquarters	118	.98	chi-square test	Girls and boys demonstrated the greatest suicide rate at 16 and 19 years, respectively. The combined suicide rate was significantly higher in the rural areas (1.48) than in Athens (0.48) and the other urban areas (0.98). Boys committed suicide more frequently than girls in Athens and other urban areas, whereas girls did so in the rural areas.	17
**14**	Guus Berkelmans, et al, 2020, Netherlands, data from 2013-2017 (59)	To understand socio-demographic risk factors in youth suicides	Retrospective	Micro-data of Statistics Netherlands	501	3.6	Chi-square test	Higher suicide rates among male youths, older youths, those of Dutch descent and youths living alone. Substantial geographical differences between provinces and healthcare regions (suicide rates among in-patients of psychiatric institutions are many times higher than average suicide rates). Background, living with their parents; months, age	16
**15**	RICHARD H, et al, 1984, United States, data from 1964-1978 (60)	To analyze the geographic pattern of this youthful suicide epidemic,	Retrospective	The 1970 US Bureau of the Census		9.6	A national interstate analysis	A strong inverse relationship between youthful suicide and population density	14
**16**	Emma Hofstra, et al,2018, Netherlands, data from 1995-2015 (61)	Trends in suicide incidence and to explore if any associations differ in relation to gender, age, and province of residence	Retrospective longitudinal population-based study	he national register of natural and unnatural deaths data, as registered by Statistics Netherlands	4658	0–19 = 1.2and 20–29 = 8.3	Poisson regression analysis	Suicide rates peak in the spring, up to 8% higher than in the summer. Suicides occurred more than twice as often in men than in women; no evidence was found of a differential effect by season in the age groups.	17
**17**	Orellana, et al, 2016, Brazil, data from 2000-2012 (62)	To examine the spatial-temporal distribution and risk of suicide in the indigenous and non-indigenous population of the Brazil	Descriptive ecological study	Information Department of the Brazilian Unified Health System	181	Non-indigenous=8.1 indigenous= 65.2	kernel analysis	The suicide risk among the indigenous population, males and villages residents was higher than in the non-indigenous population, female and rural residents.	16
**18**	Taghaddosinejad, et al, 2010, Tehran, iran, data from 2002-2006 (63)	To identify the characteristics of completed suicide by burning in Tehran	Retrospective analysis	Tehran’s Legal Medicine Organization and judiciary system	15-24 = 145	1.6	Pearson’s chi-square test, and Fisher exact test	Most victims were residents of suburban areas. Self-burning was more frequent in females than in males and was noted mainly in young age groups’ residents of suburban areas with low level of education.	17
**19**	Bradford D. Gessner, Alaska, 1997, data from 1979-1993 (64)	Geographic variations in suicide rates are associated with marriage rates, unemployment rates, per capita income, and education rates, in the youth of Alaska.	Statewide Analysis	Alaska Bureau of Vital Statistics	14-19 = 216	14-19 = 31.5 Male 14-19 = 47.5 Female 14-19 = 13.6	Knox pair method	Suicide rates varied by race, gender, and local census area of residence. Within census areas, suicide rates correlated inversely with the percentage of all households headed by a married couple.	18
**20**	Katherine Hempstead, New Jersey, 2006, data from,1999–2001 (65),	To investigate whether fatal and non-fatal self-injury exhibit similar geographic patterns	Secondary analysis	Hospital discharge data, death certificates and medical examiner data	10-24year=199	6.6	Negative binomial regression	Completed suicides have a somewhat different geographical pattern. Isolation such as low population density and high proportions of households with only one member were predictive of completed suicides, male divorce rate, percent non-Hispanic whites person, county density, municipality density, rural center, percent of households with one member, unemployment rate	16
**21**	Chia-Yueh Hsu, et al, Hong Kong, 2015, data from 2005-2010 (66),	To investigate the spatial patterning of suicide and the association of suicide rates with a broad range of area socioeconomic characteristics	Secondary analysis	Coroner’s Court	10-44 = 1639	14 male, 8 female	Moran’s I statistics, Bayesian hierarchical models	In general, suicide rates were higher in areas with higher levels of social fragmentation (except population mobility) and socioeconomic deprivation. Areas with more households living in public housing and a higher population density also showed higher suicide rates.	17
**22**	Farrell, et al, Ireland, 2015, data frome2009-2011 (23),	To examine the small area level association between suicide and the following three area level factors, deprivation, social fragmentation and population density	Secondary analysis	Irish Central Statistics Office	15-39 = 781	15	Negative binomial regression	Overall deprivation had the strongest independent effect on small-area rates of suicide, with the most deprived areas showing the greatest risk of suicide. Low population density (rurality) was associated with an increased suicide risk in males	18

### Data synthesis

Considering the method of systematic review for examining a spatial, geographic, and demographic analysis of factors associated with adolescent and youth suicide, a narrative synthesis was considered to be the most appropriate method of data analysis.

## Results

Eight studies from Europe, seven from America, six from Asia, and one from Oceania were reviewed in the present study. No studies from Africa were retrieved in the review. The lowest suicide rate was reported at 0.97 per 100,000 in Hong Kong and the highest at 65.2 per 100,000 in Brazil. Most studies were longitudinal and retrospective. Regression and Poisson analyses were the most frequently used statistical tests in the studies. The largest sample size pertained to a study from the US with 71,306 people and the smallest sample size was 118 people in a study from Greece.

### Age

Review of literature revealed lower suicide rates for the age group under 15 as compared with the age group 15-29 in most studies. As age increased in the 15-29 age group, suicide rates also increased (49, 54, 58, 60).

### Gender

In most of the reviewed studies, men had higher suicide rates than women (8, 49–51, 54, 59, 60, 63, 65), but some studies reported higher death rates due to self-burning in women aged 15-24 (64), and completed suicide rates as higher in girls living in rural areas (8, 59). Also, despite the higher suicide rates for men, certain studies showed no difference between men and women (57), especially in the under-14 age bracket (53). In one study, however, suicide rates for men and women in rural areas were about twice as high as those for men and women in urban areas (55).

### Depression

Higher suicide rates have been reported in regions with high numbers of patients with treated depression or under treatment for mental disorders (57).

### Education level

Studies reported a significant relationship between low levels of education and self-burning (63). Only one study reported a significant relationship between high levels of education in mothers and higher suicide rates in adolescents and the youth (57).

### Social isolation

Certain studies reported a low population density (60) with a high proportion of one-member and single-parent households as a suicide predictor (53, 54, 57, 65). One study showed lower suicide rates for people living in families than for those living alone (59).

### Race and ethnicity

In two studies conducted in urban areas, minority people suffered higher suicide rates than other ethnic and racial groups (49, 65). However, the majority of studies showed higher suicide rates for natives of Alaska, Brazil, the Netherlands, Hong Kong, and Ecuador than non-natives, immigrants, and white individuals (8, 49, 56, 59, 62, 64).

### Marital status

Some studies have confirmed the relationship between divorce and suicide [66]. Suicide rates also showed an inverse relationship with the percentage of households headed by married couples (65). Suicide rates were higher among widowed and divorced people than among married couples (64). Married women showed a higher rate of suicide and death by self-burning than married men (48).

### Employment status

Several studies have confirmed the relationship between unemployment and suicide (48, 54, 65). Only one study showed a higher suicide rate in regions with low unemployment (57).

### Deprivation and social fragmentation

Higher suicide rates were observed in underprivileged regions with social fragmentation (23, 66). Studies have shown higher suicide rates in men than in women in underprivileged regions (23).

### Urban and rural area and the suburbs

The majority of studies reported higher suicide rates in regions classified as rural (23, 23, 48, 49, 54, 58, 62, 62, 65). Higher suicide rates were also reported in the suburbs relative to urban areas (49, 63). Other studies reported higher suicide rates in cities with psychiatric hospitals (59). Certain studies reported higher suicide rates for rural girls and urban boys (58). Other studies reported higher suicide rates for urban girls and rural boys (23). One study on the urban areas of the US reported a higher suicide rate among men and natives compared to women and non-natives (49).

### Socioeconomic status

Higher suicide rates were observed in areas with a lower socioeconomic status (23, 66).

### Highlands, mountainous, and coastal areas

A study in Ecuador reported a high suicide rate in the highlands and mountainous areas of the Amazon (8). A higher suicide rate was also observed in the Welsh and English coastal areas (54). Higher suicide rates were observed for the youths of northern Italy and Australia compared to other regions (51, 52).

### Population density and housing

Areas with a larger number of families living in more populated public housing (66) had higher suicide rates (23, 53, 56, 66). Some studies also report higher suicide rates in low density populations (54, 60), or lower risk of suicide in high density populations (23).

### Seasons of the year

Some studies reported that suicide rates were higher in the spring (50, 61) and summer (50). Other studies, however, showed no relationship between suicide and days and seasons of the year (8, 59, 63).

### Miscellaneous

One study showed a relationship between the poverty rate, GDP per capita, employment rate, foreign married women’s rate, crime rate, number of psychiatrists, and social welfare costs, with suicide rates in 15-19-year-old adolescents (53).

## Discussion

A classical study by Durkheim found geographical and temporal variations effective in mortality from suicide and community effective in the tendency of individuals to commit suicide. This study found suicide frequency in a population to reflect its geographical and socioeconomic features, and suicide risk factors at a community level not to simply constitute the sum of individual risk factors. The limitations of the studies conducted on individual risk factors were also highlighted in this study in terms of investigating the fundamental causes and preventive measures of suicide (67). The present research aimed at exploring the spatial, geographical and demographic factors related to suicide in adolescents and the youth. Numerous studies on spatial and temporal variations in suicide reported mortality from suicide as a function of geographical location (19, 40). In contrast, Fowler and Caley reported insignificant differences in the frequency and risk of suicide in 1.3 million individuals in England and Wales among different populations and geographical locations. They explained their findings by the scarcity of suicide as an outcome and found collecting data on suicide to rarely lead to discovering local groups and targeted interventions (68).

The present systematic review showed a higher suicide frequency for the age group of 15-29 years old than that for the age group of below 15 years. Similarly, numerous studies suggest the growing suicide frequency at the age of below 29 years than in other age groups (69, 70). Research indicates positive relationships between age and suicide frequency such that 6% of suicides were reported in adolescents aged below fifteen, 34% in those aged 15-19 and 60% in the 20-24 age group (55). These findings are a global alarm to urgently adopt appropriate preventive measures. Research also relates the higher risk of suicide at lower ages to receiving decreased support, poor religious activities, living alone or in single-parent families, alcohol abuse, unemployment and facing new stressful responsibilities such as financially or vocationally supporting oneself or one’s family (69, 71–73).

In line with literature, the present systematic review found increased suicide frequencies in males than those in females of the adolescent and young age group (74, 75), which can be explained by the heavier burden of economic loads carried by men (66). Gender-based social expectations of men, their higher exposure to risk and their lower tendency to seek help during depression or on the verge of suicidal behaviors can be attributed to an emphasis on their commitment to be strong, independent and capable (8, 76). The present review rarely observed a higher suicide frequency in women than that in men; e.g. the higher suicide rate in Iranian women was attributed to their cultural background and means of suicide (63).

The present research observed a higher suicide frequency in the patients with psychological disorders, including depression. Previous studies also reported more suicidal ideation and attempts in adolescents with depression or living in areas with high suicide rates. These adolescents felt a lack of access to medical services in their neighborhood (77, 78). Promoting access to health services thus appears essential for evaluating health and preventing suicide in adolescents (57). Depression might have lowered the tendency to receive psychological services. It is therefore recommended that preventive services be actively provided for patients with depression, especially in high-risk areas.

The present research found negative relationships between education levels and suicide frequency. Low levels of literacy have also been found to relate to suicide rates in literature (79). The lower suicide frequency in educated individuals can be explained by their higher perception of the damage caused by suicide (80).

The present study found the total suicide frequency to be higher in native, racial and ethnic groups. Similarly, a higher suicide frequency was reported in the native Taiwanese (81). Research explains this finding by easy access to pesticides, especially in rural areas (81); nevertheless, the small proportion of minority populations should be included in the analysis of ethnic and racial data. The data should also be cautiously interpreted due to failure to report suicides (65).

The present research observed positive relationships between social isolation and suicide frequency. Similarly, previous studies suggest shrinking peer-to-peer networks and social isolation can increase suicide rates (74). Research also demonstrates higher suicide rates in areas with more single-parent families (81). Studies on differences in suicide rates between rural and urban areas have found environmental factors such as transition from an agricultural economy, decline in population, marital instability and growing rates of living in isolation to increase social fragmentation.

In line with the present study, research suggests positive associations between divorce and suicide rates (75, 79, 82). The risk of suicide was also found to increase in singles and divorced individuals (83). Marriage can exert its protective effects through improving socio-emotional stability and conformity to social norms. The significant and positive relationship observed between divorce and suicide, even in high-income strata, reflects the effects of social welfare on suicide (75, 84). Marriage at young ages can increase suicide frequency in women by increasing their family and social stresses (63). According to Durkheim, divorce rates, number of children, indicators of social integrity, and family ties play a key role in suicide rates. In fact, the higher the divorce rate and the fewer the children, the weaker the social integrity of the family and thus the higher the suicide rate (85).

The present findings showed relationships between suicide and unemployment. A review of the studies mostly conducted in Western countries showed that unemployment is a socioeconomic factor associated with suicide rates (83, 86). Despite the reported negative relationships of the socioeconomic status and unemployment with suicide (75, 86), these relationships have not been confirmed in the youth (57, 87). These findings appear rational given that individuals aged below 18 are not employed or allowed to be employed in most countries. Certain studies also observed no significant relationships between unemployment and suicide rates (79).

According to Durkheim, increased suicide caused by weakened social norms can be associated with rapid economic and demographic changes. Social displacement caused by population and economic expansion and contraction can create an environment for suicide in the absence of social workforce that serves to reduce suicidal tendencies. In line with this argument by Durkheim, the present and previous research suggests social solidarity constitutes a major predictor of cross-sectional and temporal changes in suicide rates (75). Research also suggests positive relationships between socioeconomic deprivation and suicide (75, 86). Furthermore, socioeconomic growth has been found to prevent or reduce suicide (88).

This study observed the positive relationships of deprivation and social disintegration with suicide in the youth and adolescents (89). Some studies have found deprivation more effective than social disintegration in suicide, whereas certain researchers reported social disintegration as the dominant factor (54, 83); nevertheless, these two variables were also found not to affect suicide elsewhere (90).

The present study found a higher suicide rate in adolescents and the youth living in rural areas and on the outskirts than in those living in urban areas; nevertheless some studies reported higher suicide frequencies in urban areas (91, 92). According to previous studies, the risk and frequency of suicide is higher in rural than in urban areas (74, 75, 81, 93) This finding can be explained by higher development, better socioeconomic status and access to psychiatric services in urban areas as compared to villages (55, 94). The limited economic infrastructure and jobs coupled with high unemployment, low education levels and economic deprivation in rural areas can adversely affect mental health. Climatic conditions, social isolation, lack of intimate friends and jobs and more firearms can be associated with higher suicide rates in rural areas (55, 94). Research suggests a spatial inequality in suicide rates between rural and urban residents (91, 92).

According to previous studies, the risk factors of suicide include social isolation, stigma of psychological disorders, easy access to poisonous pesticides, economic problems and concentration of ethnic minority groups (81, 95). In line with the present research, previous studies demonstrated a higher suicide frequency in rural than urban men (94). The higher prevalence of mortality in urban areas can be explained by the extent of deprivation, low socioeconomic status and large ethnic population in the neighborhood where suicide occurs (92, 96).

This study found higher suicide rates in areas with a low population density and coastal and mountainous regions. Similarly, research suggests negative relationships between population density and suicide rates (81). In low-density population areas, individuals at risk may receive inadequate outpatient care and treatment for psychological disorders and drug abuse compared to the services provided in urban areas. The residents of low density population areas also tend to keep and use firearms, and some studies revealed relationships between higher suicide rates and using firearms (65).

The present study observed no regular patterns of suicide; nevertheless, previous studies reported the highest suicide frequency in the spring, early summer and fall (61, 97, 98). Certain researchers have also confirmed the relationship between season and suicide in young age groups (99, 100). It appears that seasonal patterns constitute a popular factor in suicide risk and seasonal variations in mortality from suicide can help identify factors affecting or preventing suicide.

### Limitations

Inappropriate age classifications in previous studies prevented a favorable comparison and meta-analysis in some cases. The limitations of primary studies, such as the possibility of inaccurate recording of suicide statistics in some years or underreporting could have also affected the results of this study. Alongside these limitations, however, the present study also has strongpoints including that, to our knowledge, this is the first study on the systematic evaluation of spatial, geographic, and other factors related to suicide in adolescents and the youth; and its findings can serve as a guide for qualitative and quantitative research which may identify potential preventive interventions.

### Policy making implications

Developing training courses and implementing suicide prevention strategies in schools with the help of local leaders, influencers and peers; developing suicide prevention strategies in villages and low-density areas; reducing access to firearms in villages; allocating funds to geographical areas with a high prevalence of suicide among the native people; increasing access to mental health services, especially for men, individuals of 15-29 years, people living in rural areas and suburbs; socio-economic development (policies to reduce divorce, increase the level of education, reduce unemployment), and informing psychologists and social workers about spatial and geographic factors related to suicide in teenagers and young adults.

## Conclusions

Geographic and demographic variables were found more effective than spatial variables on suicide in the youth and adolescents. Mortality from suicide was higher in men, residents of rural and low population density areas, natives, 15-29 age group, individuals suffering deprivation, social disintegration and unemployment, divorced individuals and singles, single-parent families, patients with psychological disorders and individuals with low education levels. These findings suggest that policy makers take spatial and demographic factors into consideration when health systems allocate resources for suicide prevention, and that national policymakers integrate demographic and geographic variables into health service programs. Finally, future intervention studies should seriously address the role of the variables in this study in reducing the prevalence of suicide in teenagers and young adults.

## Data availability statement

All data generated or analyzed during this study are included in this published article, and the datasets used and/or analyzed during the current study available from the corresponding author on reasonable request.

## Author contributions

MG: Conceptualization, Writing – review & editing. RT: Conceptualization, Methodology, Writing – review & editing. ZM: Conceptualization, Methodology, Writing – review & editing. SK: Conceptualization, Methodology, Project administration, Writing – original draft. MF: Conceptualization, Writing – review & editing. MM: Data curation, Writing – review & editing. SAM: Conceptualization, Methodology, Writing – review & editing. SSM: Investigation, Supervision, Writing – review & editing. SA: Conceptualization, Writing – review & editing. RK: Investigation, Writing – review & editing. ND: Conceptualization, Methodology, Writing – original draft.
